# Effects of short sleep and sleep deprivation on thyroid hormones and indices: a Bayesian multilevel analysis from the ELSA-Brasil cohort

**DOI:** 10.1590/0102-311XEN203525

**Published:** 2026-08-10

**Authors:** João Ferreira Silva, Aline Araújo Nobre, Aline Silva-Costa, Isabela Martins Benseñor, Rosane Harter Griep, Maria de Jesus Mendes da Fonseca

**Affiliations:** 1 Universidade Estadual do Maranhão, São Luís, Brasil.; 2 Programa de Computação Científica, Fundação Oswaldo Cruz, Fiocruz, Rio de Janeiro, Brasil.; 3 Departamento de Epidemiologia e Bioestatística, Universidade Federal Fluminense, Niterói, Brasil.; 4 Centro de Pesquisa Clínica e Epidemiológica do Hospital Universitário, Universidade de São Paulo, São Paulo, Brasil.; 5 Instituto Oswaldo Cruz, Fundação Oswaldo Cruz, Fiocruz, Rio de Janeiro, Brasil.; 6 Escola Nacional de Saúde Pública Sergio Arouca, Fundação Oswaldo Cruz, Fiocruz, Rio de Janeiro, Brasil.

**Keywords:** Sleep Duration, Sleep Deprivation, Thyrotropin, Thyroxine, Triiodothyronine, Duração do Sono, Privação do Sono, Tireotropina, Tiroxina, Tri-Iodotironina, Duración del Sueño, Privación de Sueño, Tirotropina, Tiroxina, Triyodotironina

## Abstract

Short sleep duration and sleep deprivation are increasingly frequent in the population and associated with negative health outcomes. Few longitudinal studies have assessed the influence of sleep parameters on thyroid hormones and related indices. This study aimed to analyze the longitudinal association between short sleep duration and sleep deprivation and thyroid hormones and indices, stratified by sex. This prospective cohort analysis included 9,564 participants (4,649 men and 4,915 women) from the first (2012-2014) and second (2016-2018) follow-up waves of the *Brazilian Longitudinal Study of Adult Health* (ELSA-Brasil). Individuals using medications for thyroid dysfunction or drugs known to alter thyroid hormone levels were excluded. Data on short sleep duration, sleep deprivation, sociodemographic characteristics, and health-related behaviors were obtained from multidimensional questionnaires. Thyroid hormones were titrated in centrifuged serum samples collected after a 12-hour overnight fast using a third-generation immunoenzymatic assay. Crude and adjusted Bayesian multilevel models stratified by sex were used to estimate coefficients and 95% credibility intervals (95%CrI). In models adjusted for age, race/skin color, educational attainment, excessive alcohol consumption, smoking, level of physical activity, coffee consumption, and menopause (for women), both men and women with short sleep duration showed lower TSH values compared to those with normal sleep duration. Only women with short sleep duration showed lower TSHI and the values (coefficient.: -0.6019; 95%CrI: -0.9072; -0.2894) compared to women with normal sleep duration. Our findings emphasize the importance of adequate sleep duration and of avoiding sleep deprivation whenever possible to ensure homeostatic maintenance of thyroid hormones.

## Resumo

Sono de curta duração e a privação de sono, cada vez mais frequentes na população, estão associadas a resultados negativos de saúde. Poucos estudos longitudinais avaliam a influência dos parâmetros do sono nos hormônios e índices da tireoide. Este estudo objetivou analisar a associação longitudinal entre o sono de curta duração e a privação de sono, além dos hormônios e índices da tireoide, estratificados por gênero. Efetuou-se uma análise de coorte prospectiva com 9.564 participantes (4.649 homens e 4.915 mulheres) das primeiras (2012-2014) e segunda ondas de acompanhamento (2016-2018) do *Estudo Longitudinal de Saúde do Adulto* (ELSA-Brasil). A amostra excluiu indivíduos que usavam medicamentos para disfunção da tireoide ou que alteravam os hormônios da tireoide. Duração do sono e privação de sono, características sociodemográficas e comportamentos relacionados à saúde foram obtidos a partir de questionários multidimensionais. Hormônios da tireoide foram ajustados em amostras de soro centrifugadas após jejum noturno de 12 horas, utilizando um ensaio imunoenzimático de terceira geração. Modelos bayesianos multinível, brutos e ajustados, estratificados por gênero, foram usados para estimar coeficientes e intervalos de 95% de credibilidade (ICr95%). Em modelos ajustados por idade, raça/cor, escolaridade, consumo excessivo de álcool, tabagismo, nível de atividade física, consumo de café e menopausa (para mulheres), homens e mulheres com sono de curta duração apresentaram valores mais baixos para TSH em comparação com homens e mulheres com duração normal do sono, respectivamente. Apenas mulheres com sono de curta duração apresentaram valores mais baixos para o coeficiente TSHI (-0,6019; ICr95%: -0,9072; -0,2894) comparado à mulheres com duração normal de sono. Nossos achados enfatizam a importância de uma duração adequada do sono e de evitar a privação de sono sempre que possível afim de garantir a manutenção homeostática dos hormônios da tireoide.

## Resumen

El sueño de corta duración y la privación del sueño, cada vez más comunes en la población, se asocian con resultados negativos para la salud. Son pocos los estudios longitudinales que evalúan la influencia de los parámetros del sueño sobre las hormonas y los índices tiroideos. Este estudio tuvo como objetivo analizar la asociación longitudinal entre el sueño de corta duración y la privación del sueño, así como las hormonas y los índices tiroideos, estratificados por género. Se realizó un análisis de cohorte prospectivo con 9.564 participantes (4.649 hombres y 4.915 mujeres) de la primera (2012-2014) y la segunda olas de seguimiento (2016-2018) del *Estudio Longitudinal de Salud en Adultos* (ELSA-Brasil). La muestra excluyó a las personas que utilizaban medicamentos para la disfunción tiroidea o que alteraban sus hormonas tiroideas. La duración y privación del sueño, las características sociodemográficas y los comportamientos relacionados con la salud se obtuvieron a partir de cuestionarios multidimensionales. Se ajustaron las hormonas tiroideas en muestras de suero centrifugadas tras un ayuno nocturno de 12 horas, utilizando un inmunoensayo enzimático de tercera generación. Se utilizaron modelos bayesianos multinivel, brutos y ajustados, estratificados por género, para estimar los coeficientes e intervalos del 95% de credibilidad (ICr95%). En los modelos ajustados por edad, raza/color, escolaridad, consumo excesivo de alcohol, tabaquismo, nivel de actividad física, consumo de café y menopausia (para mujeres), los hombres y las mujeres con sueño de corta duración presentaron valores más bajos de TSH en comparación con los hombres y las mujeres con duración normal del sueño, respectivamente. Solamente las mujeres con sueño de corta duración presentaron valores más bajos para el coeficiente TSHI (-0,6019; ICr95%: -0,9072; -0,2894) en comparación con las mujeres con duración normal del sueño. Nuestros hallazgos resaltan la importancia de una duración adecuada del sueño y de evitar la privación del sueño siempre y cuando sea posible para garantizar el mantenimiento homeostático de las hormonas tiroideas.

## Introduction

Thyroid-stimulating hormone (TSH), free thyroxine (fT4), and free triiodothyronine (fT3) are central to metabolic homeostasis and in regulating energy balance and systemic metabolism ^1^. Free T4, when converted to fT3 (the biologically active form), supports the healthy development and function of the brain, heart, and other organs, while also regulating lipid metabolism and maintaining normal reproductive function ^2^.

Given the importance of thyroid hormones in metabolic regulation, alterations in sleep duration and quality may have profound endocrine consequences. Short sleep duration and sleep deprivation exert widespread effects on metabolic and endocrine processes at both molecular and systemic levels ^1^ ^,^ ^2^. These effects vary according to age, biological sex, and type of sleep disruption. Dysregulation of the hypothalamic-pituitary-thyroid (HPT) axis resulting from sleep curtailment, circadian misalignment, or sleep disruption has significant public health implications, as this axis is essential for cardiometabolic health ^1^ ^,^ ^2^ ^,^ ^3^. Importantly, women are disproportionately affected by both sleep disturbances and thyroid disorders, with potentially wide-ranging implications for health across the lifespan ^1^.

Despite the well-established physiological roles of TSH, fT4, and fT3, important gaps remain in understanding how interindividual and intraindividual variability in thyroid hormone metabolism influences long-term health outcomes ^3^. Conventional assessments based on single time-point hormone concentrations may fail to capture subtle alterations in HPT axis regulation that accumulate over time and contribute to cardiometabolic dysfunction, accelerated biological aging, and increased disease risk. In this context, more sensitive markers of thyroid axis function, such as the Thyroid-Stimulating Hormone Index (TSHI) and the Thyrotroph Thyroxine Sensitivity Index (TTSI), have emerged as valuable tools for quantifying central sensitivity and feedback regulation within the HPT axis ^4^. These indices may offer deeper insight into variability in thyroid hormone regulation and its long-term health implications, particularly under conditions of chronic sleep disruption and circadian misalignment ^5^.

The fT3/fT4 ratio reflects the efficiency of peripheral conversion of fT4 to the biologically active fT3 via deiodinase activity ^4^. This ratio has been proposed as a robust indicator of thyroid hormone metabolic variability and may serve as a stronger prognostic marker of thyroid function than isolated measurements of fT4 or fT3 ^2^.

From a chronobiological perspective, TSH exhibits both circadian and ultradian (pulsatile) rhythms, with concentrations rising in the late afternoon and early evening and peaking during the initial phases of sleep, followed by a decline overnight to reach nadir levels during daytime ^6^. Although less pronounced, fT3 also displays a circadian pattern, with peak levels occurring in the early morning hours (approximately 2:30-3:30AM) ^6^. In contrast, the presence and characteristics of a circadian rhythm for fT4 remain less well established ^6^.

Adequate sleep is therefore essential for endocrine homeostasis and the proper functioning of multiple physiological systems. Cross-sectional evidence suggests that sleep duration is not consistently associated with TSH or fT4 levels but is inversely associated with fT3 concentrations ^7^. Notably, each additional hour of sleep has been associated with an approximate reduction of 0.017pg/mL in fT3 levels in both men and women ^7^. However, because these findings are derived from a cross-sectional design, they do not clarify the temporal sequence of this association or whether changes in sleep duration precede alterations in thyroid hormone levels.

Experimental and observational studies have demonstrated that sleep deprivation or restriction induces a range of physiological responses, including increased sympathetic activity, vasoconstriction, elevated TSH levels, and acute reductions in fT4 and fT3 concentrations ^8^ ^,^ ^9^ ^,^ ^10^ ^,^ ^11^ ^,^ ^12^. However, findings regarding the impact of short sleep duration and sleep deprivation on thyroid hormone dynamics remain inconsistent, and population-based longitudinal epidemiological studies examining these associations are scarce. Moreover, sex-specific differences in sleep architecture and thyroid function may contribute to heterogeneity in the existing literature ^13^ ^,^ ^14^ ^,^ ^15^. Women generally report longer sleep duration yet exhibit a higher prevalence of sleep and thyroid disorders; they are also more morning-oriented and spend more time in non-rapid eye movement (NREM) sleep. Conversely, men tend to have later sleep timing and a higher proportion of rapid eye movement (REM) sleep ^13^ ^,^ ^14^ ^,^ ^15^. Whether these differences are independent of thyroid function or warrant sex-specific analytical approaches remains unclear.

Given these considerations, this study aims to longitudinally investigate the associations between short sleep duration, sleep deprivation, and thyroid hormone levels and indices, including TSHI, TTSI, and the fT3/fT4 ratio, stratified by sex.

## Methods

### Study design

This prospective cohort analysis used data from the *Brazilian Longitudinal Study of Adult Health* (ELSA-Brasil), conducted across five universities and one research center in six Brazilian states (Bahia, Espírito Santo, Minas Gerais, Rio Grande do Sul, São Paulo, and Rio de Janeiro). The main objective of ELSA-Brasil is to investigate the incidence and progression of chronic diseases, particularly cardiovascular diseases and diabetes.

### Context and participants

**Figure 1:**
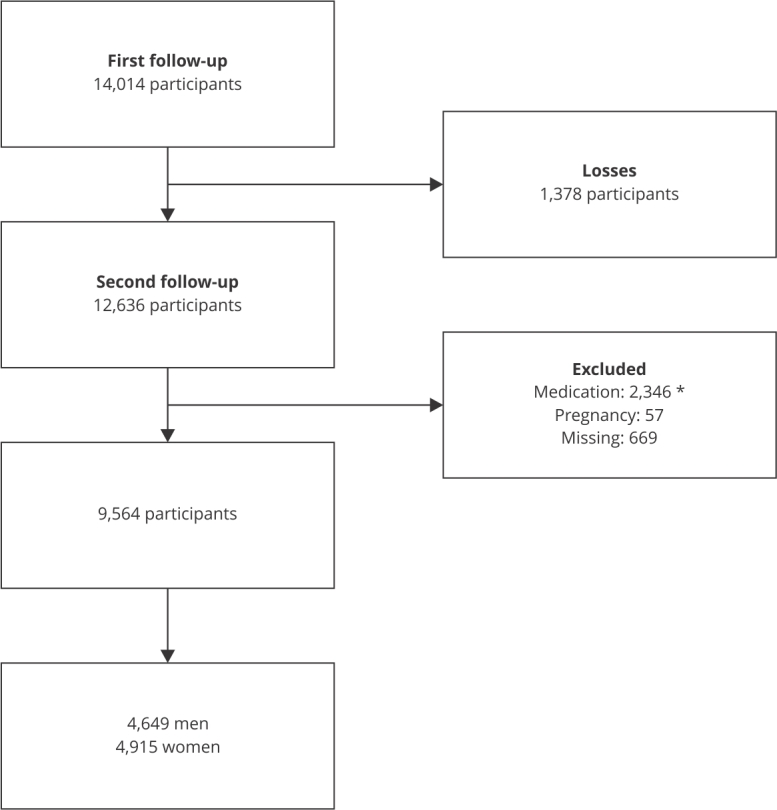
Flowchart of selected participants.

At baseline (2008-2010), 15,105 active and retired public servants aged 35-74 years (6,887 men and 8,218 women) were recruited ^16^ ^,^ ^17^. Of these, 14,014 returned for the first follow-up evaluation (2012-2014) and 12,636 for the second evaluation (2016-2018), producing the data used in the present analyses. Participants were excluded if they were taking medications known to interfere with thyroid hormone levels, including amiodarone, biotin, glucocorticoids (hydrocortisone, prednisolone, dexamethasone), antiepileptics (phenobarbital, phenytoin, primidone, divalproex sodium, valproic acid), antipsychotics (haloperidol), and other agents such as heparin, furosemide, carbidopa, levodopa, lithium, metoclopramide, and rifampicin, Individuals receiving treatment for hyperthyroidism (methimazole or propylthiouracil) or hypothyroidism (levothyroxine) were also excluded. Additionally, participants taking psychotropic medications - including antidepressants (tricyclics, selective serotonin reuptake inhibitors ENT#091;SSRIsENT#093;, serotonin-norepinephrine reuptake inhibitors ENT#091;SNRIsENT#093;, monoamine oxidase inhibitors ENT#091;MAOIsENT#093;, and atypical antidepressants), mood stabilizers, antipsychotics, tranquilizers, sedatives, or hypnotics - were excluded. Those using medications for attention-deficit/hyperactivity disorder (e.g., methylphenidate, lisdexamfetamine, amphetamine salts) or autism spectrum disorder, pregnant women, and participants with missing data on study variables were also excluded. The final analytical sample comprised 9,564 participants (4,649 men and 4,915 women), who were followed for approximately four years (Figure 1).

* Individuals were excluded if they were using medications known to interfere with thyroid function (amiodarone, biotin, glucocorticoids ENT#091;hydrocortisone, prednisolone, dexamethasoneENT#093;, antiepileptics ENT#091;phenobarbital, phenytoin, primidone, divalproex sodium, valproic acidENT#093;, haloperidol, heparin, furosemide, carbidopa, levodopa, lithium, metoclopramide, and rifampicin), as well as medications for the treatment of hyperthyroidism (methimazole or propylthiouracil) or hypothyroidism (levothyroxine). Participants were also excluded if they were using psychotropic medications, including antidepressants (tricyclics, selective serotonin reuptake inhibitors ENT#091;SSRIsENT#093;, serotonin-norepinephrine reuptake inhibitors ENT#091;SNRIsENT#093;, monoamine oxidase inhibitors ENT#091;MAOIsENT#093;, and atypical antidepressants), mood stabilizers, antipsychotics, tranquilizers, sedatives, or hypnotics, as well as medications for attention-deficit/hyperactivity disorder (e.g., methylphenidate, lisdexamfetamine, amphetamine salts) or autism spectrum disorder.

The ELSA-Brasil study was approved by the institutional review boards of the six participating institutions under protocol numbers 669/06 (University of São Paulo - USP), 343/06 (Oswaldo Cruz Foundation - Fiocruz), 041/06 (Federal University of Espírito Santo - UFES), 186/06 (Federal University of Minas Gerais - UFMG), 194/06 (Federal University of Rio Grande do Sul - UFRGS), and 027/06 (Federal University of Bahia - UFBA). All participants provided written informed consent. The current study was also approved by the Institutional Review Board of the Sergio Arouca National School of Public Health, Oswaldo Cruz Foundation (ENSP/Fiocruz, under protocol number 6.137.460, CAAE: 69771623.9.0000.5240).

### Outcome variables: thyroid hormones and indices

TSH, fT4, and fT3 levels were measured in centrifuged serum samples collected after a 12-hour overnight fast using a third-generation immunoenzymatic assay (Roche Diagnostics; https://diagnostics.roche.com/global/en/home.html). Hormones were measured during the first (2012-2014) and second (2016-2018) follow-up waves.

The target thyroid indices included the Jostel TSHI ^18^, which estimates the maximum TSH reserve in the pituitary after adjustment for negative feedback from fT4, providing a precise estimate of pituitary dysfunction severity. As a complementary indicator of pituitary function, TSHI provides a sufficiently precise and sensitive assessment of the severity of pituitary function by TSH level ^19^. TSHI is used to evaluate alterations originating outside the thyroid15. The index is calculated as follows:

TSHI=In([TSH])+0.1345×[fT 4]

Another index used to assess thyrotropic function is the TTSI, which is calculated using TSH and fT4 concentrations and the upper limit of the fT4 reference interval, denoted by limit upper (Lu) ^20^. This index represents HPT axis feedback regulation, and elevated values have been associated with thyroid hormone resistance and pituitary deregulation. It has been used to assess HPT axis deregulation due to genetic mutations and genetic factors. TTSI is calculated as follows:

$$TTSI=\frac{100\left[\mathrm{TSH}\right]\left[\mathrm{fT}4\right]}{\mathrm{Lu}}$$

We also considered the fT3/fT4 conversion ratio, which reflects thyroid function and hormone action in tissues. The hormone fT4 is converted to active fT3 in peripheral tissues and can be measured by this ratio (the lower the ratio, the worse the prognosis in thyroid dysfunctions) ^21^. The index has been shown to demonstrate greater sensitivity and specificity for autoimmune thyroid disorders compared to isolated thyroid hormone measurements ^22^. Higher fT3/fT4 ratios have also been associated with a lower risk of all-cause mortality, cardiovascular mortality, and cardiovascular disease ^23^.

### Exposure variables: short sleep duration and sleep deprivation

Exposure variables were obtained from multidimensional questionnaires administered during the first and second follow-up waves of ELSA-Brasil.

Sleep duration was measured with the question: “On average, how many hours do you sleep on a typical night?”. Responses were categorized into three groups: short duration (≤ 6 hours), normal duration (> 6 and ≤ 8 hours), and long duration (> 8 hours). Due to the small percentage of individuals with long duration (3.5%), the variable was dichotomized as short duration (≤ 6 hours) and long duration (> 6 hours) ^12^.

Sleep deprivation was assessed by calculating the difference between the desired number of hours of sleep - based on the question: “How many hours would you like to sleep to feel refreshed?” - and the self-reported number of hours slept on a typical night. Sleep deprivation was analyzed continuously as the difference between the two measures. Negative values resulting from this calculation were considered zero ^24^.

### Covariables

Selected covariables were collected during the first and second follow-up waves of ELSA-Brasil using multidimensional questionnaires and were selected for this analysis based on the literature. These included age, self-reported color/skin color (White, Mixed-race, Black, Asian, and Indigenous), educational attainment (university, secondary, or primary), smoking (non-smoker, former smoker, current smoker), excessive alcohol consumption (yes or no), level of physical activity (intense, moderate, light), coffee consumption (never, less than once a day, one to three times a day, and more than three times a day), and menopause status (yes or no, for women only).

Excessive alcohol consumption was assessed using questions on types of beverages consumed (beer, wine, *cachaça*, vodka, and whisky), frequency (daily, weekly, monthly), and amount consumed (mL/day). Excessive drinking was defined as alcohol consumption ≥ 210g/week for men and ≥ 140g/week for women ^25^.

Physical activity was assessed using the *International Physical Activity Questionnaire* (IPAQ), which estimates weekly time spent in light, moderate, and intense physical activities based on metabolic equivalents (MET) assigned to each reported activity ^26^. Physical activity level was classified as low (< 600 MET-min/week), moderate (600-3,000 MET-min/week), or vigorous (≥ 3,000 MET-min/week) ^26^. Frequency of coffee consumption was assessed using a question from the food consumption questionnaire. Responses were categorized as never/almost never, ≤ 1 time/day, 1-3 times/day, and > 3 times/day ^27^.

Menopause status was assessed using closed-ended questions regarding menstrual history. Menopause was defined as the absence of menstruation for more than six months among women in menopausal age range or those who reported natural cessation of menses.

Given that prior literature reports that sleep characteristics and thyroid hormone levels vary between men and women ^28^ ^,^ ^29^, biological sex was considered a potential effect modifier, and all analyses were stratified accordingly.

### Statistical analysis

Continuous variables were summarized as medians and interquartile ranges (IQR), while categorical variables were described as absolute and relative frequencies (%). Outcome distributions were explored using violin plots and boxplots, stratified by sex and follow-up wave of ELSA-Brasil.

To test the hypothesis that short sleep duration and sleep deprivation are associated with lower levels of TSH, TSHI, and TTSI and higher levels of fT4, fT3, and the fT4/fT3 conversion ratio, crude and adjusted Bayesian multilevel models were estimated. Models with random intercepts were specified assuming a skew-normal distribution for the outcomes to account for their asymmetry. These models are particularly suitable for longitudinal data, as they account for dependence among repeated measurements within the same individual over time, while also accommodating outcomes with negative values (such as TSHI) ^30^. The intraclass correlation coefficient (ICC) was calculated based on the mean of posterior distributions of the random-intercept variance and the residual variance ^30^ ^,^ ^31^. In skew-normal models, skewness and dispersion parameters were assumed to be constant across observations, and covariate effects were incorporated solely in the location parameter.

The Bayesian approach was chosen because of its greater robustness in settings where asymptotic assumptions may be violated. Moreover, it offers flexibility for incorporating random effects directly into the likelihood function and enables direct interpretation of credible intervals.

Results were presented as posterior means of the coefficients along with their corresponding 95% credible intervals (95%CrI), as well as the posterior probabilities of each coefficient being greater (P(β>0)) or less than zero (P(β<0)). These probabilities directly represent the evidence supporting the direction of the association, in contrast to frequentist p-values, which are based on the probability of the data under a null hypothesis.

Crude random-intercept models were fitted separately for each exposure-outcome combination, including a binary indicator for the follow-up wave. Two adjusted models were then estimated. Model 1 included age, race/skin color, and educational attainment. Model 2 additionally included smoking status, excessive alcohol consumption, physical activity level, and frequency of coffee consumption. In models restricted to women, menopausal status was also included.

Multicollinearity among covariates was assessed using variance inflation factors (VIF) and, for categorical variables, generalized VIF (GVIF) adjusted for degrees of freedom. All covariates showed low values (GVIF^(1/(2Df)) close to 1 and below 1.4), indicating no relevant multicollinearity.

Models were fitted using Bayesian inference with two Markov chains, each run for 3,000 iterations, including 1,000 warm-up iterations to avoid the effect of initial values. This resulted in 2,000 post -warm-up samples per chain and 4,000 posterior samples in total. Posterior uncertainty was summarized using equal-tailed 95%CrI, defined by the 2.5th and 97.5th percentiles of the posterior distributions. Default priors from the *brms* package were used, including non-informative priors for fixed-effect coefficients and weakly informative priors for variance and skewness parameters.

Chain convergence was assessed using the potential scale reduction factor (R̂) and the effective sample size (ESS). All parameters showed R̂ values close to 1 (typically < 1.01) and adequate Bulk_ESS and Tail_ESS values, indicating good mixing and stable estimates. No thinning was applied, as the No-U-Turn Sampler (NUTS), an adaptive Hamiltonian Monte Carlo algorithm, was used. This algorithm automatically adjusts trajectory lengths to prevent unnecessary backtracking and therefore does not require thinning to reduce autocorrelation between successive samples. Analyses were performed using Hamiltonian Monte Carlo sampling via Stan (https://mc-stan.org/), implemented through the *brms* ^32^ package in R, version 4.5.2 (http://www.r-project.org).

## Results

For both sexes, higher median levels of TSH, TSHI, and TTSI was observed in the second wave compared to the first (Table 1). Among women, fT4 and the fT3/fT4 ratio also showed higher median values in the second wave. In contrast, no differences in median fT3 levels were observed between waves (Table 1).

**Table 1:** Study variables according to follow-up wave and sex. *Brazilian Longitudinal Study of Adult Health* (ELSA-Brasil), 2012-2018.

Variables	Women				Men			
	First follow-up		Second follow-up		First follow-up		Second follow-up	
Continuous variables	Median	IQR	Median	IQR	Median	IQR	Median	IQR
TSH (mIU/L)	1.88	1.34	2.07	1.55	1.95	1.38	2.15	1.61
fT4 (ρmol/L)	14.93	2.45	15.06	2.70	15.32	2.70	15.32	2.96
fT3 (ρmol/L)	4.61	0.77	4.61	0.61	4.92	0.61	4.92	0.77
TSHI	11.41	10.45	12.81	10.91	12.15	10.72	13.72	10.96
fT3/fT4	0.30	0.06	0.31	0.06	0.32	0.06	0.32	0.07
TTSI	122.50	87.50	135.31	100.23	129.46	92.80	143.63	108.25
Sleep deprivation	2.00	2.00	1.30	2.00	1.00	2.00	1.00	2.00
Age	54.00	13.00	58.00	12.00	54.00	13.00	58.00	13.00
Categorical variables	n	%	n	%	n	%	n	%
Sleep duration (hours)								
> 6	2,433	49.50	2,433	49.50	2,183	47.00	2,232	48.00
≤ 6	2,482	50.50	2,482	50.50	2,466	53.00	2,417	52.00
Educational attainment								
Primary	380	7.70	380	7.70	611	13.10	611	13.10
Secondary	1,652	33.60	1,652	33.60	1,429	30.70	1,429	30.70
University	2,883	58.70	2,883	58.70	2,609	56.10	2,609	56.10
Race/Skin color								
White	2,451	49.90	2,451	49.90	2,437	52.40	2,437	52.40
Mixed-race	1,314	26.70	1,314	26.70	1,433	30.80	1,433	30.80
Black	957	19.50	957	19.50	632	13.60	632	13.60
Asian	161	3.30	161	3.30	89	1.90	89	1.90
Indigenous	32	0.70	32	0.70	58	1.20	58	1.20
Excessive alcohol consumption								
Yes	418	8.50	515	10.50	1083	23.30	1216	26.20
No	4,497	91.50	4,400	89.50	3,566	76.70	3,433	73.80
Smoking								
Never	3,184	64.80	3,263	66.40	2,506	53.90	2,550	54.90
Former smoker	1,221	24.80	1,207	24.60	1,617	34.80	1,652	35.50
Current smoker	510	10.40	445	9.10	526	11.30	447	9.60
Physical activity								
Light	3,854	78.40	3,597	73.20	3,245	69.80	2,982	64.10
Moderate	785	16.00	981	20.00	884	19.00	1,086	23.40
Intense	276	5.60	337	6.90	520	11.20	581	12.50
Coffee consumption								
Never	396	8.10	396	8.10	371	8.00	371	8.00
≤ 1/day	434	8.80	434	8.80	547	11.80	547	11.80
1-3/day	2,773	56.40	2,773	56.40	2,201	47.30	2,201	47.30
> 3/day	1,312	26.70	1,312	26.70	1,530	32.90	1,530	32.90
Menopause								
Yes	3,297	67.10	4,087	83.20	-	-	-	-
No	1,618	32.90	828	16.80	-	-	-	-

fT4: free thyroxin; fT3: free triiodothyronine; fT3/fT4: thyroxin triiodothyronine conversion index; IQR: interquartile range; TSH: thyroid-stimulating hormone; TSHI: Thyroid-Stimulating Hormone Index; TTSI: Thyrotrope Sensitivity Index.

Women exhibited higher median sleep deprivation in the first wave compared to the second. The proportion of women with short sleep duration was 50.5% in both waves. Among men, 53% had short sleep duration in the first wave and 52% in the second (Table 1).

**Figure 2:**
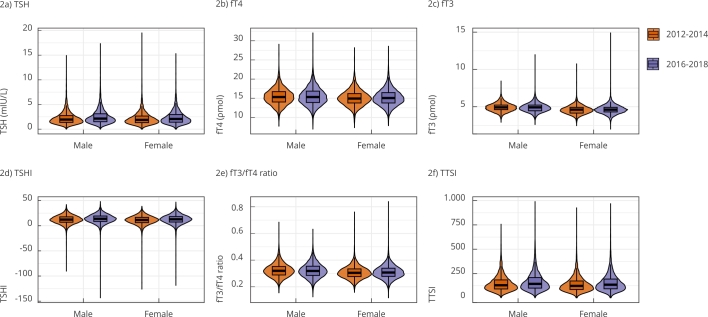
Violin plots of thyroid hormones and indices stratified by follow-up wave and sex. *Brazilian Longitudinal Study of Adult Health* (ELSA-Brasil), 2012-2018.

The distribution of outcomes was compared using violin plots and boxplots stratified by follow-up wave and sex. A positive asymmetrical distribution was observed for TSH, TTSI, fT4, fT3, and the fT3/fT4 ratio, whereas TSHI exhibited a negative asymmetrical distribution (Figure 2). The violin plots indicated similar distributions of thyroid markers between men and women across both follow-up waves. TSH exhibited wide dispersion with long tails, although median values remained stable over time. Free T4 levels were concentrated around 12-20ρmol/L, with a slight increase in the second follow-up, while fT3 levels showed little variation (4-6ρmol/L) and a similarly subtle rise. The TSHI index displayed greater variability, including negative values, but no relevant differences between groups. The fT3/fT4 ratio remained stable (~0.25-0.5), and TTSI presented the greatest variability, although with comparable medians across waves. Overall, changes between follow-ups were minimal, and patterns were consistent across sexes, with notable outliers particularly for TSH, TSHI, and TTSI (Figure 2).

fT4: free thyroxin; fT3: free triiodothyronine; fT3/fT4: free thyroxin triiodothyronine conversion index; TSH: thyroid-stimulating hormone; TSHI: Thyroid-Stimulating Hormone Index; TTSI: Thyrotrope Sensitivity Index.

The ICCs for the outcomes ranged from 0.44 to 0.71 in men and from 0.40 to 0.66 in women, indicating that a substantial proportion of the total variance was attributable to between-individual differences. These findings support the use of multilevel modeling to account for within-participant correlation across repeated measurements. ICC values for each outcome are presented alongside estimated coefficients in Tables 2 and 3.

According to our results, men with short sleep duration showed lower levels of TSH (coefficient: -0.0418; 95%CrI: -0.0807; -0.0028) and TTSI (coefficient: -2.6905; 95%CrI: -5.2036; -0.1589) compared to men with normal sleep duration. This corresponds to an average reduction of 0.04mIU/L in TSH and 0.27 in TTSI. For TSHI, there was a high posterior probability (97%) that the coefficient was negative. We also observed inverse associations between sleep deprivation and TSH, TSHI, and TTSI, with high posterior probabilities (89%, 90%, and 93%, respectively) of coefficients less than zero; however, the 95%CrI included zero (Table 2). Prospectively, target sleep parameters appeared to be inversely associated with fT4 and directly associated with fT3 and fT3/fT4, although evidence of association was observed only in the crude model for fT3 and fT3/fT4 (Table 2).

**Table 2:** Bayesian multilevel models of the relationship between sleep parameters and thyroid hormones and indices in men. *Brazilian Longitudinal Study of Adult Health* (ELSA-Brasil), 2012-2018.

	TSH (mIU/L)			fT4 (ρmol/L)			fT3 (ρmol/L)		
	β	95%CrI	P(β<0)	β	95%CrI	P(β<0)	β	95%CrI	P(β>0)
Short sleep duration									
Crude	-0.0532	-0.0909; -0.0146	0.99	-0.0136	-0.0912; 0.0649	0.64	0.0217	-0.0002; 0.0444	0.97
Adjusted *	-0.0453	-0.0828; -0.0065	0.99	-0.0057	-0.0860; 0.0726	0.55	0.0164	-0.0057; 0.0389	0.93
Adjusted **	-0.0418	-0.0807; -0.0028	0.98	-0.0067	-0.0827; 0.0711	0.57	0.0152	-0.0067; 0.0377	0.92
ICC	0.71			0.63			0.44		
Sleep deprivation									
Crude	-0.0168	-0.0299; -0.0039	0.99	-0.0129	-0.0399; 0.0145	0.83	0.0150	0.0073; 0.0229	1.00
Adjusted *	-0.0108	-0.0242; 0.0025	0.94	-0.0094	-0.0351; 0.0166	0.75	0.0044	-0.0034; 0.0122	0.87
Adjusted **	-0.0115	-0.0250; 0.0015	0.95	-0.0104	-0.0365; 0.0163	0.78	0.0037	-0.0040; 0.0114	0.83
ICC	0.71			0.63			0.44		
	TSHI			fT3/fT4			TTSI		
	β	95%CrI	P(β<0)	β	95%CrI	P(β>0)	β	95%CrI	P(β<0)
Short sleep duration									
Crude	-0.3825	-0.6758; -0.0959	0.99	0.0017	-0.0003; 0.0033	0.94	-3.4669	-5.9533; -0.9586	0.99
Adjusted *	-0.3091	-0.6040; -0.0171	0.98	0.0011	-0.0008; 0.0031	0.88	-2.8657	-5.4802; -0.4201	0.99
Adjusted **	-0.2932	-0.5918; 0.0162	0.97	0.0011	-0.0008; 0.0029	0.88	-2.6905	-5.2036; -0.1589	0.98
ICC	0.68			0.63			0.67		
Sleep deprivation									
Crude	-0.1096	-0.2129; -0.0099	0.98	0.0011	0.0005; 0.0017	1.00	-1.1691	-2.0811; -0.2168	0.99
Adjusted *	-0.0609	-0.1625; 0.0390	0.89	0.0005	-0.0002; 0.0011	0.93	-0.6559	-1.5637; 0.2464	0.92
Adjusted **	-0.0641	-0.1652; 0.0354	0.89	0.0004	-0.0002; 0.0010	0.90	-0.6965	-1.5913; 0.2044	0.93
ICC	0.68			0.63			0.67		

95%CrI: 95% credibility interval; β: estimated coefficient; fT4: free thyroxin; fT3: free triiodothyronine; fT3/fT4: free thyroxin triiodothyronine conversion index; ICC: intraclass correlation coefficients; P(β>0): posterior probability of the coefficient being greater than zero; P(β<0): posterior probability of the coefficient being less than zero; TSH: thyroid-stimulating hormone; TSHI: Thyroid-Stimulating Hormone Index; TTSI: Thyrotrope Sensitivity Index.

* Adjusted for age, race/skin color, and educational attainment;

** Adjusted for age, race/skin color, educational attainment, excessive alcohol consumption, smoking, level of physical activity, and coffee consumption.

Among women with short sleep duration, we observed lower levels of TSH (coefficient: -0.0619; 95%CrI: -0.0981; -0.0272), TSHI (coefficient: -0.6019; 95%CrI: -0.9072; -0.2894), and TTSI (coefficient: -4.0606; 95%CrI: -6.4310; -1.7386) compared to those with normal sleep duration. Specifically, the model estimated an average reduction of 0.06mIU/L in TSH, 0.60 in TSHI, and 4.1 in TTSI among women with short sleep duration compared to the reference group. Additionally, for each hour of sleep deprivation, there was an average reduction of approximately 0.1 in TSHI (coefficient: 0.0997; 95%CrI: -0.1931; -0.0050). Among women, target sleep parameters did not show meaningful associations with fT4, fT3, or fT3/fT4 (Table 3).

**Table 3:** Bayesian multilevel models of the relationship between sleep parameters and thyroid hormones and indices in women. *Brazilian Longitudinal Study of Adult Health* (ELSA-Brasil), 2012-2018.

	TSH (mIU/L)			fT4 (ρmol/L)			fT3 (pmol/L)		
	β	95%CrI	P(β<0)	β	95%CrI	P(β<0)	β	95%CrI	P(β>0)
Short sleep duration									
Crude	-0.0778	-0.1150; -0.0399	1.00	-0.0047	-0.0800; 0.0674	0.56	0.0131	-0.0097; 0.0353	0.88
Adjusted *	-0.0658	-0.1022; -0.0295	0.99	-0.0131	-0.0872; 0.0593	0.63	0.0118	-0.0099; 0.0340	0.85
Adjusted **	-0.0619	-0.0981; -0.0272	0.99	-0.0184	-0.0899; 0.0520	0.69	0.0081	-0.0132; 0.0299	0.77
ICC	0.61			0.59			0.40		
Sleep deprivation									
Crude	-0.0160	-0.0279; -0.0040	0.99	-0.0254	-0.0489; -0.0022	0.98	0.0033	-0.0036; 0.0104	0.83
Adjusted *	-0.0083	-0.0203; 0.0028	0.92	-0.0147	-0.0380; 0.0084	0.89	-0.0000	-0.0072; 0.0068	0.50
Adjusted **	-0.0071	-0.0185; 0.0044	0.88	-0.0170	-0.0409; 0.0056	0.93	-0.0015	-0.0084; 0.0053	0.35
ICC	0.61			0.59			0.40		
	TSHI			fT3/fT4			TTSI		
	β	95%CrI	P(β<0)	β	95%CrI	P(β>0)	β	95%CrI	P(β<0)
Short sleep duration									
Crude	-0.6735	-0.9633; -0.3694	1.00	0.0011	-0.0006; 0.0029	0.90	-5.0093	-7.4456; -2.6257	1.00
Adjusted *	-0.5928	-0.8878; -0.2989	1.00	0.0013	-0.0004; 0.0030	0.94	-4.2990	-6.8331; -1.8574	1.00
Adjusted **	-0.6019	-0.9072; -0.2894	1.00	0.0011	-0.0006; 0.0029	0.91	-4.0606	-6.4310; -1.7386	1.00
ICC	0.66			0.53			0.59		
Sleep deprivation									
Crude	-0.1636	-0.2592; -0.0727	1.00	0.0008	0.0003; 0.0014	1.00	-1.1942	-1.9576; -0.4333	1.00
Adjusted *	-0.0987	-0.1931; -0.0050	0.98	0.0004	-0.0001; 0.0010	0.95	-0.5584	-1.3393; 0.2242	0.93
Adjusted **	-0.0892	-0.1827; 0.0007	0.97	0.0004	-0.0001; 0.0009	0.93	-0.5151	-1.3065; 0.2249	0.91
ICC	0.66			0.53			0.59		

95%CrI: 95% credibility interval; β: estimated coefficient; fT4: free thyroxin; fT3: free triiodothyronine; fT3/fT4: free thyroxin triiodothyronine conversion index; ICC: intraclass correlation coefficients; P(β>0): posterior probability of the coefficient being greater than zero; P(β<0): posterior probability of the coefficient being less than zero; TSH: thyroid-stimulating hormone; TSHI: Thyroid-Stimulating Hormone Index; TTSI: Thyrotrope Sensitivity Index.

* Adjusted for age, race/skin color, and educational attainment;

** Adjusted for age, race/skin color, educational attainment, excessive alcohol consumption, smoking, level of physical activity, coffee consumption, and menopause.

## Discussion

This study aimed to longitudinally analyze associations between short sleep duration, sleep deprivation, and thyroid hormones and indices, stratified by sex, over four years of follow-up in a large cohort of highly educated Brazilian public servants. To or knowledge, this is the first large prospective study to demonstrate the impact of sleep parameters on thyroid biomarkers and indices in both men and women. Men and women with short sleep duration exhibited lower TSH and TTSI values compared to those with normal sleep duration. Additionally, women with short sleep duration showed lower TSHI values relative to those with adequate sleep duration. The results suggest a potential novel mechanism: short sleep duration may act directly on the pituitary and secondarily at the thyroid level, rather than the reverse. Specifically, the findings indicate a possible reduction in the sensitivity of thyrotropes to the stimulation of the hypothalamus, a brain structure that receives afferent inputs from the suprachiasmatic nucleus and is responsible for sleep regulation ^33^ ^,^ ^34^.

A recent cross-sectional study by Wang et al. ^7^ investigated the association between sleep duration and thyroid function in 8,102 North Americans. In crude analyses, a statistically significant association was observed between sleep duration (analyzed as a continuous variable) higher TSH levels ^7^. The authors ^7^ observed similar results when sleep duration was categorized into three groups (< 7 hours, 7-9 hours, > 9 hours), results that are consistent with those observed in our study. However, the authors did not stratify their analyses by sex, which hinders comparison with our findings. As discussed by the authors, the lower TSH levels observed among individuals with short sleep duration may be explained by increased sympathetic activity and circadian disruption, conditions commonly present in individuals who sleep fewer hours ^7^.

In contrast to our findings, a study with a cross-sectional and longitudinal design ^35^ conducted among 287 men and women recruited from a hospital in western China investigated whether poor sleep quality, assessed using the *Pittsburgh Sleep Quality Index* (PSQI), was associated with elevated TSH levels. The study showed that poor sleep quality was associated with increased odds of high TSH concentrations. In the subsequent phase, individuals with high TSH levels were followed for 10 weeks and reassessed for thyroid function and sleep quality. The authors found that the proportion of TSH normalization was higher among participants who improved their sleep quality (85.4%) compared to those who continued to report poor sleep quality (6.4%) ^35^. Importantly, similar associations were observed after excluding individuals without autoimmune thyroiditis, indicating that the association between poor sleep quality and elevated TSH levels appears to be independent of autoimmune thyroiditis. The authors suggest that the direct inhibitory influence of negative sleep aspects on TSH secretion may represent the main underlying mechanism ^35^.

Furthermore, unlike our results, an experimental before-and-after study with 118 participants of both sexes found a significant increase in TSH levels and concomitant reductions in fT4 and fT3 concentrations after three days of sleep deprivation (four hours of nighttime sleep) ^11^. The authors also found that TSH, fT4, and fT3 values returned to baseline eight days after recovering sleep ^11^.

Evidence suggests that increased TSH levels in individuals with short sleep duration or sleep deprivation are more frequently detected in cross-sectional or short-term experimental studies. In contrast, as observed in our results, longitudinal analysis appear to indicate a reduction in TSH over time ^8^. One possible explanation relates to the chronicity of exposure to sleep disturbances. There is an acute TSH elevation in the initial days of exposure until there is a thyroxin (fT4) response, culminating in the reduction in TSH levels over time ^33^.

Short sleep duration also appears to increase central sensitivity to thyroid hormones, reflected by lower TSHI values in women and lower TTSI values in both sexes. These indices represent the sensitivity of pituitary thyrotropes to thyroid hormone-mediated negative feedback; lower values indicate greater central sensitivity ^3^.

A recent study ^3^ assessed the association between sleep duration and thyroid function indices (TSHI and TTSI, referred to as TT4SI in the research) in 1,918 North Americans. The authors ^3^ reported that for each additional hour of sleep, there was a 0.232 increase (95% confidemce interval - 95%CI: 0.120; 0.343) in TSHI and 0.014 (95%CI: 0.007; 0.020) in TTSI. Although sleep duration was analyzed continuously, the effects were similar to ours, as individuals with short sleep duration in our study showed lower values for both indicators.

The main clinical implication of our findings is that adverse target sleep parameters may contribute to dysregulation of the HPT axis, especially thyrotropes in the anterior pituitary. This is supported by the observed increase in sensitivity to thyroid hormones, expressed as lower TSHI and TTSI values. Such alterations may represent a key mechanism underlying the long-term reduction in TSH levels ^36^.

The strengths of this study include its original proposal, given the scarcity of longitudinal studies on this topic, and its large sample size. Thyroid hormones, exposures, and confounders were measured at two time points, and the longitudinal design enabled prospective inferences regarding the impact of sleep parameters on outcomes. Another advantage was the use of a Bayesian model, which enabled modeling outcome asymmetry via a skew-normal distribution and interpreting credible intervals as probabilities. Furthermore, the availability of data on medication in the cohort facilitated the exclusion of individuals using medicines for thyroid dysfunctions, drugs known to interfere with thyroid function, and psychotropics.

Despite its strengths, this study has some limitations, such as subjective measurement of sleep parameters. However, subjective evaluation remains the most feasible and commonly used method for assessing sleep characteristics in large cohorts given the logistical complexity and high costs associated with gold-standard measurements ^37^. Another limitation was the small proportion (3.5%) of individuals reporting long sleep duration (> 8 hours), which prevented analyzing the effect of this stratum on the outcomes.

## Conclusion

The associations identified in this study suggest that sleep duration may influence the production and secretion of TSH, an important marker of thyroid function in both sexes. These findings are particularly relevant to women’s health, as short sleep duration was also associated with lower TSH-related indices in this group. Moreover, both sleep parameters assessed appear to affect peripheral and central sensitivity to thyroid hormones. Our results highlight the importance of adequate sleep as a key component of health promotion. Sleep disruption may contribute to dysregulation of metabolic hormones that are essential for overall health. From a clinical perspective, assessment of sleep habits should be considered when interpreting thyroid function tests, as improving sleep duration may represent a feasible and non-pharmacological strategy to support endocrine health.

## Data Availability

The sources of information used in the study are indicated in the body of the article.
